# Supplementary material for: Modeling glioblastoma heterogeneity as a dynamic network of cell states
Source: Mol Syst Biol. 2021 Sep 16;17(9):e10105. doi: 10.15252/msb.202010105 (PMC8444284; doi:10.15252/msb.202010105)
Supplement: Supplementary file 6 — Source Data for Figure 5 [file MSB-17-e10105-s004.zip › Figure5A_sourcedata/GSEA_3071/nosigdb_stateIII.GseaPreranked.1622795155562/OLSEN_MESENCHYMAL.html]

Details for gene set OLSEN\_MESENCHYMAL[GSEA]

|  || Dataset | state33071 |
| Phenotype | NoPhenotypeAvailable |
| Upregulated in class | na\_neg |
| GeneSet | OLSEN\_MESENCHYMAL |
| Enrichment Score (ES) | -0.60408735 |
| Normalized Enrichment Score (NES) | -3.3529267 |
| Nominal p-value | 0.0 |
| FDR q-value | 0.0 |
| FWER p-Value | 0.0 |
Table: GSEA Results Summary

  

Fig 1: Enrichment plot: OLSEN\_MESENCHYMAL      
 Profile of the Running ES Score & Positions of GeneSet Members on the Rank Ordered List

  

| PROBE | GENE SYMBOL | GENE\_TITLE | RANK IN GENE LIST | RANK METRIC SCORE | RUNNING ES | CORE ENRICHMENT || 1 | RPS25 |  |  | 19 | 0.527 | 0.0096 | No |
| 2 | RPLP1 |  |  | 25 | 0.514 | 0.0225 | No |
| 3 | MT2A |  |  | 31 | 0.499 | 0.0350 | No |
| 4 | S100A11 |  |  | 48 | 0.464 | 0.0437 | No |
| 5 | RPL32 |  |  | 55 | 0.457 | 0.0548 | No |
| 6 | FTL |  |  | 67 | 0.445 | 0.0642 | No |
| 7 | SERF2 |  |  | 111 | 0.403 | 0.0642 | No |
| 8 | S100A6 |  |  | 172 | 0.360 | 0.0587 | No |
| 9 | LGALS1 |  |  | 230 | 0.323 | 0.0529 | No |
| 10 | RPL41 |  |  | 265 | 0.303 | 0.0525 | No |
| 11 | TMSB4X |  |  | 273 | 0.300 | 0.0589 | No |
| 12 | MYL9 |  |  | 293 | 0.293 | 0.0621 | No |
| 13 | TPT1 |  |  | 324 | 0.285 | 0.0623 | No |
| 14 | TAGLN |  |  | 407 | 0.257 | 0.0482 | No |
| 15 | THY1 |  |  | 472 | -0.253 | 0.0386 | No |
| 16 | ID3 |  |  | 880 | -0.273 | -0.0590 | No |
| 17 | PRRX1 |  |  | 1032 | -0.281 | -0.0903 | No |
| 18 | FSTL1 |  |  | 1254 | -0.293 | -0.1393 | No |
| 19 | AEBP1 |  |  | 1763 | -0.322 | -0.2616 | No |
| 20 | TIMP1 |  |  | 2038 | -0.337 | -0.3231 | No |
| 21 | PLAC9 |  |  | 2086 | -0.341 | -0.3259 | No |
| 22 | SERPINF1 |  |  | 2245 | -0.352 | -0.3570 | No |
| 23 | SERPINH1 |  |  | 2921 | -0.410 | -0.5201 | No |
| 24 | EGR1 |  |  | 2961 | -0.415 | -0.5187 | No |
| 25 | EMP1 |  |  | 2998 | -0.420 | -0.5164 | No |
| 26 | PPIC |  |  | 3045 | -0.425 | -0.5165 | No |
| 27 | CD63 |  |  | 3258 | -0.452 | -0.5588 | No |
| 28 | ITM2B |  |  | 3314 | -0.463 | -0.5603 | No |
| 29 | C1R |  |  | 3364 | -0.473 | -0.5598 | No |
| 30 | ZFP36L1 |  |  | 3487 | -0.503 | -0.5775 | No |
| 31 | CTHRC1 |  |  | 3591 | -0.544 | -0.5891 | Yes |
| 32 | RRBP1 |  |  | 3595 | -0.545 | -0.5748 | Yes |
| 33 | COL14A1 |  |  | 3609 | -0.553 | -0.5629 | Yes |
| 34 | IGFBP7 |  |  | 3611 | -0.554 | -0.5478 | Yes |
| 35 | FOS |  |  | 3621 | -0.561 | -0.5347 | Yes |
| 36 | PRSS23 |  |  | 3658 | -0.584 | -0.5279 | Yes |
| 37 | COL4A2 |  |  | 3664 | -0.586 | -0.5130 | Yes |
| 38 | COL6A2 |  |  | 3674 | -0.595 | -0.4988 | Yes |
| 39 | DCN |  |  | 3723 | -0.633 | -0.4938 | Yes |
| 40 | COL5A2 |  |  | 3726 | -0.636 | -0.4767 | Yes |
| 41 | VCAN |  |  | 3741 | -0.660 | -0.4621 | Yes |
| 42 | TIMP3 |  |  | 3755 | -0.677 | -0.4467 | Yes |
| 43 | LIMA1 |  |  | 3774 | -0.706 | -0.4319 | Yes |
| 44 | PCOLCE |  |  | 3777 | -0.708 | -0.4129 | Yes |
| 45 | SPARC |  |  | 3811 | -0.759 | -0.4004 | Yes |
| 46 | BGN |  |  | 3812 | -0.761 | -0.3794 | Yes |
| 47 | LHFPL6 |  |  | 3814 | -0.762 | -0.3586 | Yes |
| 48 | COL5A1 |  |  | 3815 | -0.764 | -0.3375 | Yes |
| 49 | COL4A1 |  |  | 3829 | -0.791 | -0.3190 | Yes |
| 50 | FN1 |  |  | 3850 | -0.842 | -0.3010 | Yes |
| 51 | LUM |  |  | 3861 | -0.870 | -0.2795 | Yes |
| 52 | COL6A1 |  |  | 3863 | -0.872 | -0.2557 | Yes |
| 53 | NEAT1 |  |  | 3880 | -1.005 | -0.2321 | Yes |
| 54 | POSTN |  |  | 3885 | -1.025 | -0.2048 | Yes |
| 55 | IGFBP5 |  |  | 3895 | -1.082 | -0.1773 | Yes |
| 56 | COL6A3 |  |  | 3900 | -1.171 | -0.1460 | Yes |
| 57 | COL1A1 |  |  | 3902 | -1.191 | -0.1134 | Yes |
| 58 | COL1A2 |  |  | 3906 | -1.278 | -0.0788 | Yes |
| 59 | COL3A1 |  |  | 3918 | -1.418 | -0.0425 | Yes |
| 60 | MGP |  |  | 3927 | -1.633 | 0.0005 | Yes |
Table: GSEA details [plain text format]

  

Fig 2: OLSEN\_MESENCHYMAL: Random ES distribution      
 Gene set null distribution of ES for **OLSEN\_MESENCHYMAL**
